# Outcomes and Resource Utilization in Hyperkalemic Emergency Department Patients Treated With Patiromer or Sodium Zirconium Cyclosilicate

**DOI:** 10.1016/j.acepjo.2025.100158

**Published:** 2025-05-10

**Authors:** William Frank Peacock, Zubaid Rafique, Julie Gayle, James Neuenschwander, Harold Brown, Jennifer Kammerer, Jeffrey Budden, Ning Rosenthal

**Affiliations:** 1 Department of Emergency Medicine, Baylor College of Medicine, Houston, Texas, USA; 2 PINC AI Applied Sciences, Premier Inc, Charlotte, North Carolina, USA; 3 Department of Emergency Medicine, Doctors Hospital, Columbus, Ohio, USA; 4 U.S. Medical Affairs, CSL Vifor, Redwood City, California, USA

**Keywords:** hyperkalemia, patiromer, sodium zirconium cyclosilicate, emergency department, potassium binders, health care resource utilization

## Abstract

**Objectives:**

Hyperkalemia (HK) is a life-threatening condition commonly treated in emergency department (ED). Real-world outcomes associated with ED administration of potassium binders are limited. This study assessed ED discharge disposition and resource utilization in hyperkalemic ED patients treated with patiromer or sodium zirconium cyclosilicate (SZC).

**Methods:**

We performed a retrospective cohort study using discharge data from 230 hospitals in the Premier PINC AI Healthcare Database. ED patients were included if they were ≥18 years old, had potassium ≥5 mEq/L, and received patiromer/SZC during January 1, 2019 to December 31, 2021. Descriptive statistics and multivariable logistic regression analysis were used to compare outcomes between binder cohorts.

**Results:**

Of 18,248 patients meeting selection criteria, 6480 and 11,768 received patiromer and SZC, respectively. Compared with patients receiving SZC, patients receiving patiromer were older, more likely to be White and Hispanic, with Medicare as primary insurance, and had a higher mean Charlson-Deyo Comorbidity Index. Adjusted analysis showed that the odds of being admitted to an inpatient ward were similar between cohorts (adjusted odds ratio [aOR] = 1.09; 95% CI: 0.96, 1.23). Patients receiving patiromer had lower mortality during index visit (12.0% vs 16.3%; aOR = 0.85; 95% CI: 0.76, 0.95), but higher odds of all-cause hospitalization (3.0% vs 0.8%; aOR = 3.54; 95% CI: 2.73, 4.59) and hyperkalemia-related hospitalization (1.1% vs 0.3%; aOR = 3.28; 95% CI: 2.15, 5.00) during 30-day follow-up than patients receiving SZC.

**Conclusion:**

This large nationally representative sample of US ED patients showed that patiromer and SZC treatment had similar discharge dispositions to inpatient ward/home. Patients receiving patiromer had lower in-hospital mortality. Patients receiving SZC had lower 30-day all-cause and hyperkalemia-related hospitalization risks.

**The Bottom Line**

Newer potassium binders, including patiromer and sodium zirconium cyclosilicate, are equally effective and safe in treating patients with hyperkalemia in the emergency department setting, and both are associated with less adverse events than sodium polystyrene sulfonate.

## 1 Introduction

### 1.1 Background

Acute hyperkalemia (HK) is a life-threatening condition.^1^, ^2^, ^3^, ^4^ Although there is no standard definition, it is commonly defined as elevated serum potassium (K^+^) concentrations >5.0 mEq/L or 5.5 mEq/L.^4^ The prevalence of HK in the general US population is estimated between 1.57% and 2.7%,^5^^,^^6^ and it is even more prevalent in persons with chronic kidney disease (CKD), heart failure (HF), diabetes, and hypertension.^5^^,^^7^ Patients with multiple comorbidities are at even greater risk of HK, with observed prevalence between 6.35% and 9.3%.^5^^,^^7^^,^^8^ Approximately 50% of all patients with CKD or HF will have a HK event, making these conditions important risk factors.^5^^,^^9^ Finally, HK can be drug induced due to utilization of renin-angiotensin-aldosterone system inhibitors (RAASi) and other medications that impact the kidney’s ability to remove K^+^.^1^^,^^4^^,^^10^^,^^11^

### 1.2 Importance

Prevalence of HK among general patients visiting the US emergency department (ED) was reported to be between 3.6% and 4%.^12^^,^^13^ Immediate treatment of acute HK is important in an ED setting because higher levels of serum K^+^ are associated with increased morbidity and mortality.^4^^,^^10^^,^^14^

A variety of treatments are given for the management of acute HK, including potassium-binding drugs, dialysis, and temporizing agents (albuterol, insulin, bicarbonates, calcium [chloride/gluconate], loop diuretics, and dextrose).^15^ Novel K^+^ potassium binders (KB) such as patiromer and sodium zirconium cyclosilicate (SZC) are effective in treating patients with HK in the ED.^16^, ^17^, ^18^ These newer binders have a shorter and more predictable time until onset of action compared with their long-standing competitor, sodium polystyrene sulfonate (SPS).^9^ In addition, SPS is associated with multiple gastrointestinal side effects including vomiting, diarrhea, nausea, and in rare cases, colonic necrosis and death.^9^^,^^19^ A phase 2, randomized, double-blind, placebo-controlled study of 70 patients showed that SZC may result in a bigger reduction in serum K^+^ level within 2 hours of administration to patients with HK than placebo.^16^ In a separate study, a single 25.2 g dose of patiromer significantly lowered serum K^+^ within 2 hours after administration to hyperkalemic patients in the ED.^17^ Although both of the newer binders are efficacious, there are limited data on potential differences, if any, in clinical outcomes and health care resource utilization between patiromer and SZC for treating patients with HK in the ED.^20^

### 1.3 Goals of This Investigation

This study sought to assess the difference in discharge disposition, clinical outcome (ie, in-hospital mortality), and health care resource utilization (ie, 30-day risk of all-cause and HK-related hospitalization) between patients with HK treated with either patiromer or SZC in the ED.

## 2 Methods

### 2.1 Study Design and Setting

We performed a retrospective cohort study using Health Insurance Portability and Accountability Act (HIPAA)-compliant, statistically deidentified data from the Premier PINC AI Healthcare Database (PHD). Serum K^+^ laboratory values were assessed using the general laboratory data available in the PHD.

The PHD is a large, geographically diverse, hospital discharge database that contains date-stamped, service-level, all-payer data from inpatient, and hospital-based outpatient visits.^21^ As of December 2023, there were over 1.4 billion inpatient and outpatient (including ED) discharges from over 1300 hospitals in 45 US states and the District of Columbia since 2000. The PHD contains data from standard hospital discharge files, including a patient’s demographics, disease states, medications, surgical procedures, laboratory tests, diagnostic and therapeutic services, as well as costs and charges for each service provided. In addition, information on hospital characteristics, including geographic location, bed size, and teaching status is available. Finally, 30-day outcome data are available if patients return to a participating PHD institution.

The PHD data has been rigorously validated at both facility and visit level. Extensive data validation occurs during implementation and with each subsequent (monthly) data submission by each hospital. Before data can be published into the PHD, thresholds on cases, charges, and cost must be met. These are reviewed by Premier monthly. Premier also performs a reconciliation, comparing data a hospital has submitted to the hospital’s financial statement. Data must meet reconciliation variance requirements to be published into the PHD. These steps occur at each time when each Premier team member submits data to Premier. If issues are identified in the database, Premier works with the hospitals impacted to update and correct the data in the database. The extensive data validation process is consistently applied to all PHD hospitals to ensure data are as accurate and complete as possible.^22^ The PHD provides a unique source of real-world data to conduct evidence-based and population-based analyses of drugs, devices, other treatments, disease states, epidemiology, resource utilization, health care economics, and clinical outcomes. The PHD data has been widely used by academia, industry and government agencies (eg, the Centers for Disease Control and Prevention, the National Institute of Health, and the United States Food and Drug Administration) for conducting real-world evidence. studies with over 1000 peer-reviewed publications.^22^, ^23^, ^24^, ^25^, ^26^, ^27^, ^28^, ^29^, ^30^, ^31^, ^32^, ^33^

All PHD information is deidentified and compliant with the HIPAA. Based on US Title 45 Code of Federal Regulations, Part 46, because the study used existing deidentified hospital discharge data and recorded information could not be identified directly or through identifiers linked to individuals, this study was exempt from institutional review board approval, and no informed consent of study participants was pursued because of the nature of the deidentified data. This study followed the Strengthening the Reporting of Observational Studies in Epidemiology (STROBE) reporting guideline.

### 2.2 Selection of Participants

All patients who presented to an ED between January 1, 2019 and December 31, 2021, and who met the following criteria were included in the study: aged ≥ 18 years at index visit, had a principal or secondary discharge diagnosis of HK (International Classification of Disease, Tenth Revision, Clinical Modification [ICD-10-CM] code of E87.5) at their index visit, had serum K^+^ values between 5 and 8 mEq/L during the time spent in ED, and received patiromer or SZC in the ED. Patients were excluded if they received more than one type of KB, were pregnant, or were seen in hospitals without continuous data submission during the 180-day look-back and 30-day follow-up periods. Considering the look-back and follow-up periods, the complete study time frame was from July 1, 2018 through March 31, 2022.

### 2.3 Outcomes

The primary outcome of interest was ED discharge disposition, which was categorized as being admitted to any inpatient ward, being discharged to home, being discharged to other facilities, ED expiration, or having an unknown disposition status. Secondary outcomes included in-hospital mortality during index visit, and health care resource use such as 30-day all-cause hospitalization, and 30-day HK-related hospitalization during 30-day follow-up post index discharge.

The main exposure was KB use during the ED index visit, which was dichotomized as patiromer versus SZC. Patient demographics (eg, age, race, sex, ethnicity, and primary payor type), hospital characteristics (eg, teaching status, urban rural status, bed size, and geographic location), and visit characteristics (eg, admission point of origin) were assessed. Clinical characteristics and comorbid conditions were measured at the index visit and during the 180-day look-back period. The Charlson-Deyo Comorbidity Index (CCI) was used to assess baseline comorbidities. The presence of each condition and the CCI total score for each patient were reported. Clinical conditions assessed included acute kidney injury (AKI), cardiac arrhythmia, HF (acute, chronic, or any), hypertension, ischemic heart disease, morbid obesity, trauma, burn, crash, dialysis during index visit, dialysis during 45 days before index visit, CKD (mild vs severe), COVID-19 infection, and medications that included SGLT2i and nonbinder temporizing treatments received in ED during index visit. KB dosing and K^+^ levels were assessed during index visit. ICD-10-CM, Current Procedural Terminology, and Healthcare Common Procedure Coding System codes were used to define clinical and comorbid conditions. Specific definitions can be found in Tables S2 and S3).

### 2.4 Analysis

We first compared the patient demographic and clinical characteristics, visit and hospital characteristics, and key study outcomes between patients receiving patiromer and those receiving SZC at ED during index visit, using descriptive statistics. Continuous variables are presented as mean with SD when normally distributed, and as median, 25th and 75th percentiles (ie, Q1 and Q3) when skewed, with minimum and maximum values presented for all parameters. Categorical data were expressed as counts and percentages of patients in the categories. We used *t* test to assess the difference in continuous variables that were normally distributed between comparison groups and Wilcoxon rank-sum test to determine differences when comparing groups in which continuous data were not normally distributed. Chi-square tests were used for categorical variables. Missing data were recoded as unknown for categorical data. For continuous variables, patients with missing records were excluded from the analysis.

Multivariable logistic regression modeling was used to determine if independent associations existed in primary (discharge disposition) and secondary outcomes (30-day all-cause hospitalization risk, 30-day HK-related hospitalization risk, and in-hospital mortality) between patiromer and SZC patient groups, while controlling for demographic, visit, hospital, clinical characteristics, non-KB treatments, and K^+^ values. The final list of covariates was determined based on backward selection. Only those covariates that were statistically significant at 0.05 level, or known confounders based on prior knowledge, were included in the final regression model. Statistical significance was set at 0.05 for all comparisons in this study.

All analyses were performed using R v.3.6.3 (R Foundation for Statistical Computing).

## 3 Results

### 3.1 Patient, Hospital, and Visit Characteristics

A total of 18,248 patients (patiromer: 6480; SZC: 11,768) meeting the selection criteria were included in the analysis (see Table S1). Compared with the SZC cohort, the patiromer cohort was older (mean age, 68.1 vs 67.1 years; *P* = .0458); more likely to be White (66.3 vs 62.1%; *P* < .0001); Hispanic (8.7% vs 5.5%; *P* < .0001); and had Medicare as primary health insurance (71.1% vs 67.9%; *P* < .0001). Patients receiving patiromer were more likely to be treated in large (48.2% vs 39.1%), nonteaching (57.3% vs 39.3%), rural (14.7% vs 9.8%) hospitals, as well as hospitals in the south (53.0% vs 46.5%), than patients receiving SZC, respectively (all *P* < .0001). A higher percentage of patients receiving patiromer were admitted from nonhealth care origins (eg, from home) than patients receiving SZC (93.4% vs 88.5%; *P* < .0001) (Table 1).

**Table 1:** Baseline characteristics of emergency department patients with hyperkalemia, stratified by type of potassium binder treatment (N = 18,248).

Characteristics	Patiromer		SZC		*P* value
Total number of patients (%)	6480	35.5%	11,768	64.5%	
Patient demographics					
Age					
Mean-SD	68.1	15	67.1	15	.0458
Median (Q1-Q3)	69	(59-79)	68	(58-78)	
Age					
18-39 y	288	4.4%	571	4.9%	.0025
40-59 y	1383	21.3%	2673	22.7%	
60-74 y	2489	38.4%	4621	39.3%	
75+ y	2320	35.8%	3903	33.2%	
Sex					
Male	3673	56.7%	6778	57.6%	.2319
Female	2807	43.3%	4990	42.4%	
Race					
White	4298	66.3%	7305	62.1%	<.0001
Black	1557	24.0%	2959	25.1%	
Asian	85	1.3%	223	1.9%	
Other	540	8.3%	1281	10.9%	
Ethnicity					
Hispanic	566	8.7%	645	5.5%	<.0001
Non-Hispanic	5659	87.3%	10,515	89.4%	
Unknown	255	3.9%	608	5.2%	
Health insurance status					
Medicare	4608	71.1%	7987	67.9%	<.0001
Medicaid	653	10.1%	1296	11.0%	
Private insurance	912	14.1%	1869	15.9%	
Uninsured	182	2.8%	290	2.5%	
Other/unknown	125	1.9%	326	2.8%	
Hospital characteristics					
Hospital size					
1-299	1934	29.8%	3038	25.8%	<.0001
300-499	1422	21.9%	4126	35.1%	
500+	3124	48.2%	4604	39.1%	
Teaching status					
Teaching	2770	42.7%	7144	60.7%	<.0001
Nonteaching	3710	57.3%	4624	39.3%	
Population served					
Urban	5529	85.3%	10,610	90.2%	<.0001
Rural	951	14.7%	1158	9.8%	
Geographic region					
South	3433	53.0%	5471	46.5%	<.0001
Northeast	1737	26.8%	1869	15.9%	
Midwest	501	7.7%	1883	16.0%	
West	9	0.1%	406	3.5%	
Unknown	800	12.3%	2139	18.2%	
Admission point of origin					
Nonhealth care (eg, home)	6053	93.4%	10,419	88.5%	<.0001
Clinic	126	1.9%	243	2.1%	
Transfer from an acute care hospital	122	1.9%	643	5.5%	
Transfer from long-term care or rehab facility	172	2.7%	419	3.6%	
Other/unknown	7	0.1%	44	0.4%	
Clinical characteristics					
Charlson comorbidities					
Myocardial infarction	1208	18.6%	2208	18.8%	.8414
Congestive heart failure	2887	44.6%	4848	41.2%	<.0001
Peripheral vascular disease	766	11.8%	1481	12.6%	.1328
Cerebrovascular disease	718	11.1%	1587	13.5%	<.0001
Dementia	618	9.5%	1024	8.7%	.0591
Chronic pulmonary disease	2176	33.6%	3894	33.1%	.5009
Rheumatic disease	207	3.2%	400	3.4%	.4608
Peptic ulcer disease	198	3.1%	365	3.1%	.8632
Mild liver disease	196	3.0%	348	3.0%	.7974
Diabetes with chronic complication	3081	47.5%	5220	44.4%	<.0001
Diabetes without chronic complication	729	11.2%	1426	12.1%	.0822
Hemiplegia or paraplegia	110	1.7%	263	2.2%	.0141
Renal disease	3909	60.3%	5772	49.0%	<.0001
Moderate/severe liver disease	281	4.3%	534	4.5%	.5287
Any malignancy	953	14.7%	1700	14.4%	.6324
Metastatic solid tumor	452	7.0%	690	5.9%	.003
AIDS/HIV	61	0.9%	90	0.8%	.2077
Charlson-Deyo CCI score categories					
0	309	4.8%	807	6.9%	<.0001
1-4	3067	47.3%	5974	50.8%	
≥5	3104	47.9%	4987	42.4%	
CCI score					
Mean-SD	4.6	2.8	4.2	2.8	<.0001
Median (Q1-Q3)	4.0	(3.0-6.0)	4.0	(2.0-6.0)	
Other clinical conditions					
Acute kidney injury	4412	68.1%	7687	65.3%	.0002
Cardiac arrhythmia	890	13.7%	1427	12.1%	.0018
Hypertension	4669	72.1%	7878	66.9%	<.0001
Ischemic heart disease	2799	43.2%	5010	42.6%	.4169
Morbid obesity	1528	23.6%	2770	23.5%	.9492
Trauma	65	1.0%	153	1.3%	.0772
Burn	13	0.2%	16	0.1%	.2941
Crash	24	0.4%	59	0.5%	.2082
COVID-19 infection	703	10.8%	2146	18.2%	<.0001
Chronic kidney disease severity					
Mild (stage I-IV)	2941	45.4%	4866	41.3%	<.0001
Severe (stage V and end-stage renal disease)	1193	18.4%	1917	16.3%	.0003
HF					
HF (any)	2699	41.7%	4554	38.7%	<.0001
Acute	210	3.2%	338	2.9%	.1627
Chronic	888	13.7%	1587	13.5%	.6807
Unspecified	2571	39.7%	4350	37.0%	.0003
Dialysis at index visit					
Hemodialysis	1285	19.8%	2103	17.9%	.0011
Peritoneal dialysis	891	13.8%	1638	13.9%	.7517
Dialysis history (45 d before index visit)					
Hemodialysis	910	14.0%	1400	11.9%	<.0001
Peritoneal dialysis	212	3.3%	361	3.1%	.4496
Nonbinder treatment in ED					
Albuterol	2424	37.4%	4526	38.5%	.161
Insulin	1141	17.6%	3562	30.3%	<.0001
Diuretics	3496	54.0%	6102	51.9%	.0066
Bicarbonates	2673	41.2%	4807	40.8%	.5973
Calcium chloride or calcium gluconate	2467	38.1%	4957	42.1%	<.0001
Dextrose	2583	39.9%	5494	46.7%	<.0001
SGLT2i at index visit	161	2.5%	326	2.8%	.2519
Total no. of nonbinder treatment in ED					
Mean-SD	1.88	1.24	2.04	1.35	<.0001
Median (Q1-Q3)	2	(1-3)	2	(1-3)	
Minimum-Maximum	0	5	0	5	
Highest potassium laboratory value in ED					
Mean-SD	5.94	0.59	5.97	0.61	.0002
Median (Q1-Q3)	5.8	(5.5-6.2)	5.8	(5.5-6.3)	
Minimum-Maximum	5	8	5	8	

CCI, Charlson comorbidity index; ED, emergency department; HF, heart failure; SZC, sodium zirconium cyclosilicate; SGLT2i, sodium-glucose cotransporter 2 inhibitor.

### 3.2 Clinical characteristics and comorbid conditions

As shown in Table 1, the most common Charlson-Deyo comorbidities for both groups were congestive HF (44.6% vs 41.2%), diabetes with chronic complication (47.5% vs 44.4%), and renal disease (60.3% vs 49.0%) with higher prevalence observed among patients receiving patiromer than those receiving SZC (all *p* values < .001). Accordingly, prevalence of patients with CCI score ≥5 (47.9% vs 42.4%) and mean (4.6 vs 4.2; *P* < .001) CCI score were also higher among patients receiving patiromer than those receiving SZC. Similarly, patients receiving patiromer also had higher prevalence of AKI (68.1% vs 65.3%), cardiac arrhythmia (13.7% vs 12.1%), hypertension (72.1% vs 66.9%), HF (41.7% vs 38.7%), and preindex hemodialysis (14.0% vs 11.9%) than patients receiving SZC. For nonbinder treatment, both groups had a median of 2 (q1-q3: 1-3) HK stabilizing/temporizing treatments at ED. The means of highest K^+^ laboratory values during the index ED visit were also comparable between the groups (5.94 vs 5.97) (see Table 1).

### 3.3 Outcomes

The crude prevalence of patients admitted to inpatient ward was slightly higher in those receiving patiromer than those receiving SZC, 91.9% vs 91.0% (*P* = .0187), respectively. However, after adjusting for known confounders, there is no difference in the odds of being admitted to the inpatient ward (adjusted odds ratio [aOR] = 1.09; 95% CI: 0.96, 1.23) between comparison groups. Both crude and adjusted 30-day risks of all-cause (3.0% vs 0.8%; *P* < .0001; aOR, 3.54; 95% CI: 2.73, 4.59) and HK-related (1.1% vs 0.3%; *P* < .0001; aOR, 3.28; 95% CI: 2.15, 5.00) hospitalization were higher among patients receiving patiromer than those receiving SZC. In-hospital mortality during the index visit was lower in patients receiving patiromer than in those receiving SZC (12.0% vs 16.3%; *P* < .0001). The adjusted odds of in-hospital mortality was 15% lower in patients receiving patiromer (aOR, 0.85; 95% CI: 0.76, 0.95) (Table 2).

**Table 2:** Multivariable logistic regression analysis results for primary and secondary outcomes.

Outcomes	Unadjusted			Adjusted Patiromer (vs SZC)	
	Patiromer N = 6480	SZC N = 11,768	*P* value	OR (95% CI)	*P* value
Discharge disposition (being admitted to inpatient ward vs not)^a^	5956 (91.9%)	10,710 (91.0%)	.0187	1.09 (0.96-1.23)	.2025
In-hospital mortality^b^	777 (12.0%)	1922 (16.3%)	<.0001	0.85 (0.76-0.95)	<.01
30-d all-cause hospitalization^a^	195 (3.0%)	99 (0.8%)	<.0001	3.54 (2.73-4.59)	<.0001
30-d HK-related hospitalization^a^	72 (1.1%)	37 (0.3%)	<.0001	3.28 (2.15-5.00)	<.0001

HK, hyperkalemia; OR, odds ratio; SZC, sodium zirconium cyclosilicate.

a Adjusted for patient characteristics (age, sex, race, and payer); hospital characteristics (teaching status, hospital bed size, geographic region, and population served); and clinical characteristics (hypertension, renal disease, cerebrovascular disease, metastatic solid tumor, dementia, trauma, diabetes, acute kidney injury [AKI], index dialysis, dialysis history, CKD severity, SGLT2i, cardiac arrhythmia, COVID-19 infection, insulin, albuterol, bicarbonates, calcium [chloride/gluconate], loop diuretics and highest potassium laboratory value, and Charlson Comorbidity Index score).

b Adjusted for patient characteristics (age, sex, race, and payer); hospital characteristics (teaching status, hospital bed size, geographic region, and population served); and clinical characteristics (hypertension, renal disease, cerebrovascular disease, metastatic solid tumor, dementia, trauma, diabetes, AKI, index dialysis, dialysis history, CKD severity, SGLT2i, cardiac arrhythmia, COVID-19 infection, insulin, albuterol, bicarbonates, calcium [chloride/gluconate], loop diuretics and highest potassium laboratory value, and Charlson Comorbidity Index score and discharge disposition).

## 4 Limitations

This study may be subject to several limitations. First, as any study using an administrative database, many of our conclusions are dependent on accurate and complete coding by each hospital or health system. However, it must be considered that while coding and misclassification errors are likely to have occurred, they are also likely to equally affect our comparison groups. Second, although we included all known and measurable confounders in our adjusted analysis, there could be unknown confounders biasing the relationship between binder use and key outcomes. Third, because only patients’ visits to the same hospital or health system can be linked, the capture of postdischarge outcomes may be underestimated. However, similar to coding errors, we expect such underestimates would be similar between the comparison groups. Fourth, our analysis was limited to patients with lab results and may not represent all patients who received a KB in the ED. Fifth, our analysis is limited by its inability to determine actual KB dose, or the potential for multiple doses. How this could impact outcomes cannot be discerned by our investigation. Finally, although we could determine the rates of ED therapeutic non-KB interventions known to result in the acute decrease of K^+^ concentrations as a function of intracellular K^+^ shifts (eg, albuterol, insulin/glucose, etc), and expected to be associated with improved acute outcomes, we could not identify the dosage given to individual patients. Thus, we cannot account for the potential of lower doses in the SZC group being responsible for the differences in acute mortality. Future prospective studies, controlling for dosage administration of nonbinder HK therapy, will be required to validate our findings.

## 5 Discussion

As one of the largest evaluations to date assessing outcome differences between 2 new KBs, this large, real-world, retrospective cohort study found that patients with HK receiving either patiromer or SZC in the ED had similar discharge dispositions. Although both groups had similar adjusted odds of hospitalization, the patients receiving patiromer had a higher risk of 30-day all-cause and HK-related readmissions but a lower risk of dying during index visit.

Overall, patients with HK are known to have high prevalence of AKI, CKD, hypertension, HF, and diabetes.^5^^,^^7^ Our patients had a similar clinical profile as expected for a cohort presenting to an ED for treatment of acute HK.^5^^,^^34^^,^^35^ The most common conditions we observed in our population were hypertension (68.4%), AKI (65.8%), CKD (59.3%), renal disease (53.0%), diabetes (45.5%), and congestive HF (42.0%). It is important to note that patients receiving patiromer in this sample had more severe disease comorbidities than those receiving SZC, as defined by both rates of occurrence and number of comorbidities, as indicated by the CCI.

Compared with other similar smaller reports, our inpatient admission rate is much higher. An ED HK study by Davis et al,^8^ reported a 5.8% immediate admission rate in a cohort of 6222 ED patients. This may be a function of oversampling inpatient visits with ED admission in the PHD. The adjusted odds of being admitted to inpatient wards were similar between patients given patiromer and SZC, which implies that patients treated with patiromer and SZC in the ED have similar ED discharge disposition.^11^^,^^23^ In this analysis, patients receiving SZC had lower odds for both 30-day all-cause and HK-related hospitalizations than those receiving patiromer. Rates in both groups were lower than those reported in Davis et al’s^8^ study. This could be because we only captured hospitalization if it occurred in the same hospital or health system. Higher rates of and more severe comorbidities in the patiromer group, compared with the SZC cohort, may explain why patients receiving patiromer were more likely to return to the hospital within the 30-day follow-up period. Of patients admitted to hospitals after index ED visit, patients receiving patiromer had lower in-hospital mortality, even though they had more complex baseline health conditions relative to patients receiving SZC.

In our study, patients receiving patiromer showed more favorable outcomes for in-hospital mortality during the index visit even though they had more comorbidities relative to patients receiving SZC, as defined by overall CCI and by numbers of comorbidities. The in-hospital mortality rates in our study were higher than those reported by Davis et al^8^ (10.6% for patients with K^+^ >6 mEq/L) in a US ED patient population but lower than that reported in Wu et al’s^36^ study (19.2%) conducted among ED patients with HK from an ED in Beijing, China. The reason why patiromer patients had relatively lower in-hospital mortality rate during index visit than the SZC patients could potentially be due to better management of HK status in the patiromer group and unknown confounders such as different KB dosing, but it remains largely unknown. More studies are needed to corroborate the association.

Newer KBs used to treat acute HK are known to be effective and are a reasonable option because of a better safety profile, increased tolerance, improved efficacy, and overall lower acquisition costs compared with SPS.^9^ Although much of the published literature seeks to address the effectiveness of binders in lowering serum K^+^ for patients experiencing a HK event, there remains a great need to assess additional outcomes associated with these binders. Beyond the outcomes of interest in this study, additional research is needed to address gaps in information, utility, and outcomes associated with various KB treatments.^37^

In summary, after adjusting for baseline demographic and clinical conditions, we found that patiromer and SZC treatment in the ED had similar ED discharge dispositions. However, patients receiving patiromer had a lower in-hospital mortality rate, whereas those treated with SZC had lower 30-day all-cause and HK-related hospitalizations. These results should be considered when clinicians are selecting hyperkalemia therapy for their patients. More research is needed to further define the use of these newer binders to treat acute HK.

## Author Contributions

All authors contributed extensively to the study design, study finding interpretations, and manuscript review. JG, HB, and NR had access to the study data and conducted the data analysis.

## Funding and Support

CSL Vifor provided funding for this study.

## Data Sharing

The data that support the findings of this study are available from Premier Inc, but restrictions apply to the availability of these data, which were used under license for the current study, and so are not publicly available. Data are however available from the authors on reasonable request and with permission by contacting Premier Inc (https://www.pinc-ai.com/applied-sciences).

## Conflict of Interest

WFP received research grants from Abbott, Brainbox, Quidel, Roche, Siemens; served as a consultant to Abbott, AstraZeneca, Biocogniv, Brainbox, Bristol Meyers Squibb, Instrument Labs, Janssen, Osler, Quidel, Roche, Siemens, Spinchip, CSL Vifor; and held stock/ownership of AseptiScope Inc, Brainbox Inc, Biocogniv, Inc, Braincheck Inc, Coagulo Inc, Comprehensive Research Associates LLC, Comprehensive Research Management Inc, Emergencies in Medicine LLC, Fast Inc, Forrest Devices, Ischemia DX LLC, Lucia Inc, Prevencio Inc, RCE Technologies, ROMTech, ScPharma, Trivirum Inc, and Upstream Inc. ZR reports grants and personal fees from AstraZeneca; grants and personal fees from CSL Vifor, outside the submitted work. JN serves as a consultant and on the speaker bureau at AstraZeneca, Janssen Pharmaceutical, ThermoFisher, Fisher, and Paykel; a consultant at Pfizer Inc; a speaker at Abbott; a research consultant at Siemens, Bridgesource, and CSL Behring; and owner of Asceptoscope. JG, HB, and NR are stockholders of Premier Inc. JK and JB are stockholders of CSL Vifor.

## References

[1] Hollander-Rodriguez J.C., Calvert J.F. (2006). Hyperkalemia. Am Fam Physician.

[2] Crawford A.H. (2014). Hyperkalemia: recognition and management of a critical electrolyte disturbance. J Infus Nurs.

[3] Luo J., Brunelli S.M., Jensen D.E., Yang A. (2016). Association between serum potassium and outcomes in patients with reduced kidney function. Clin J Am Soc Nephrol.

[4] Lindner G., Burdmann E.A., Clase C.M. (2020). Acute hyperkalemia in the emergency department: a summary from a Kidney Disease: Improving Global Outcomes conference. Eur J Emerg Med.

[5] Betts K.A., Woolley J.M., Mu F., McDonald E., Tang W., Wu E.Q. (2018). The prevalence of hyperkalemia in the United States. Curr Med Res Opin.

[6] Mu F., Betts K.A., Woolley J.M. (2020). Prevalence and economic burden of hyperkalemia in the United States Medicare population. Curr Med Res Opin.

[7] Lakkis J.I., Weir M.R. (2018). Hyperkalemia in the hypertensive patient. Curr Cardiol Rep.

[8] Davis J., Israni R., Betts K.A. (2022). Real-world management of hyperkalemia in the emergency department: an electronic medical record analysis. Adv Ther.

[9] Cañas A.E., Troutt H.R., Jiang L. (2023). A randomized study to compare oral potassium binders in the treatment of acute hyperkalemia. BMC Nephrol.

[10] Collins A.J., Pitt B., Reaven N. (2017). Association of serum potassium with all-cause mortality in patients with and without heart failure, chronic kidney disease, and/or diabetes. Am J Nephrol.

[11] Neuenschwander J.F., Silverstein A.R., Teigland C.L. (2023). The increased clinical and economic burden of hyperkalemia in Medicare patients admitted to long-term care settings. Adv Ther.

[12] Arampatzis S., Funk G.C., Leichtle A.B. (2013). Impact of diuretic therapy-associated electrolyte disorders present on admission to the emergency department: a cross-sectional analysis. BMC Med.

[13] Singer A.J., Thode H.C., Peacock W.F. (2017). A retrospective study of emergency department potassium disturbances: severity, treatment, and outcomes. Clin Exp Emerg Med.

[14] Kovesdy C.P. (2015). Management of hyperkalemia: an update for the internist. Am J Med.

[15] Rafique Z., Peacock F., Armstead T. (2021). Hyperkalemia management in the emergency department: an expert panel consensus. J Am Coll Emerg Physicians Open.

[16] Peacock W.F., Rafique Z., Vishnevskiy K. (2020). Emergency potassium normalization treatment including sodium zirconium cyclosilicate: a phase II, randomized, double-blind, placebo-controlled study (ENERGIZE). Acad Emerg Med.

[17] Rafique Z., Liu M., Staggers K.A., Minard C.G., Peacock W.F. (2020). Patiromer for treatment of hyperkalemia in the emergency department: a pilot study. Acad Emerg Med.

[18] Esteban-Fernández A., Ortiz Cortés C., López-Fernández S. (2022). Experience with the potassium binder patiromer in hyperkalaemia management in heart failure patients in real life. ESC Heart Fail.

[19] Harel Z., Harel S., Shah P.S., Wald R., Perl J., Bell C.M. (2013). Gastrointestinal adverse events with sodium polystyrene sulfonate (Kayexalate) use: a systematic review. Am J Med.

[20] Lemoine L., Le Bastard Q., Batard E., Montassier E. (2021). An evidence-based narrative review of the emergency department management of acute hyperkalemia. J Emerg Med.

[21] PINC AI Applied Sciences, Premier Inc (2024). https://offers.pinc-ai.com/PINC-AI-Healthcare-Database-White-Paper-LP.html.

[22] Altin S.E., Parise H., Hess C.N. (2023). Long-term patient outcomes after femoropopliteal peripheral vascular intervention in patients with intermittent claudication. JACC Cardiovasc Interv.

[23] Amin A., Moon R., Agiro A. (2022). In-hospital mortality, length of stay, and hospitalization cost of COVID-19 patients with and without hyperkalemia. Am J Med Sci.

[24] Begier E., Rosenthal N.A., Gurtman A., Kartashov A., Donald R.G.K., Lockhart S.P. (2021). Epidemiology of invasive *Escherichia coli* infection and antibiotic resistance status among patients treated in US hospitals: 2009-2016. Clin Infect Dis.

[25] Bhatt A.S., Jering K.S., Vaduganathan M. (2021). Clinical outcomes in patients with heart failure hospitalized with COVID-19. JACC Heart Fail.

[26] Cunningham J.W., Vaduganathan M., Claggett B.L. (2020). Clinical outcomes in young US adults hospitalized with COVID-19. JAMA Intern Med.

[27] Ernst F.R., Mills J.R., Berner T., House J., Herndon C. (2015). Opioid medication practices observed in chronic pain patients presenting for all-causes to emergency departments: prevalence and impact on health care outcomes. J Manag Care Spec Pharm.

[28] Kadri S.S., Gundrum J., Warner S. (2020). Uptake and accuracy of the diagnosis code for COVID-19 among US hospitalizations. JAMA.

[29] Molina R.L., Tsai T.C., Dai D. (2022). Comparison of pregnancy and birth outcomes before vs during the COVID-19 pandemic. JAMA Netw Open.

[30] Moon R.C., Rosenthal N.A. (2022). In-hospital mortality among hospitalized coronavirus disease 2019 patients in the United States: how did it change in 2021?. Open Forum Infect Dis.

[31] Palmer P.P., Walker J.A., Patanwala A.E., Hagberg C.A., House J.A. (2017). Cost of intravenous analgesia for the management of acute pain in the emergency department is substantial in the United States. J Health Econ Outcomes Res.

[32] Rosenthal N., Cao Z., Gundrum J., Sianis J., Safo S. (2020). Risk factors associated with in-hospital mortality in a US national sample of patients with COVID-19. JAMA Netw Open.

[33] Sosa M.P., McNicholas D.G., Bebla A.B. (2023). Ninety-day all-cause emergency room use among coronary artery bypass grafting patients associated with near-infrared fluorescence imaging: a retrospective cohort study. Ann Med Surg (Lond).

[34] Gorriz J.L., D'Marco L., Pastor-González A. (2022). Long-term mortality and trajectory of potassium measurements following an episode of acute severe hyperkalaemia. Nephrol Dial Transplant.

[35] Ronksley P.E., Tonelli M., Manns B.J. (2017). Emergency department use among patients with CKD: a population-based analysis. Clin J Am Soc Nephrol.

[36] Wu Y., Fu Y., Tang H. (2023). Treatment and factors associated with prognosis of hyperkalemia in the emergency department. Zhonghua Wei Zhong Bing Ji Jiu Yi Xue.

[37] Chaitman M., Dixit D., Bridgeman M.B. (2016). Potassium-binding agents for the clinical management of hyperkalemia. P T.

