# Supplementary material for: Outcomes and Resource Utilization in Hyperkalemic Emergency Department Patients Treated With Patiromer or Sodium Zirconium Cyclosilicate
Source: J Am Coll Emerg Physicians Open. 2025 May 10;6(4):100158. doi: 10.1016/j.acepjo.2025.100158 (PMC12139450; doi:10.1016/j.acepjo.2025.100158)
Supplement: Tables S1-S3 [file mmc1.docx]

**Supplemental Tables**

**Table S1. Patient Identification and Selection**

| **Study Population** | **Discharges** | **Excluded Discharges** | **Discharges Remained** | |
| --- | --- | --- | --- | --- |
| **Step 1** |  |  |  | |
| Emergency Department (ED) visit with a principal or secondary discharge diagnosis of hyperkalemia OR ED visit with potassium binder use | 1,195,449 |  |  | |
| **Step 2** |  |  |  | |
| Index discharges with a diagnosis of HK or potassium binder use | 914,800 | 280,649 | 914,800 | |
| **Step 3** |  |  |  | |
| Exclude patients without billing reconciliation table records | 912,996 | 1,834 | 912,966 | |
| **Step 4** |  |  |  | |
| Exclude pregnant patients | 911,840 | 1,126 | 911,840 | |
| **Step 5** |  |  |  | |
| Exclude patients from hospitals with no continuous data submission during the 180-day look-back and 30-day follow-up period | 906,880 | 4,960 | 906,880 | |
| **Step 6** |  |  |  | |
| Exclude patients with multiple potassium binder use | | 883,001 | 23,879 | 883,001 |
| **Step 7** |  |  |  | |
| Exclude patients with no laboratory data | 162,683 | 720,318 | 162,683 | |
| **Step 8** |  |  |  | |
| Exclude patients with SPS use or no KB use | 20,058 | 142,625 | 20,058 | |
| **Step 9** |  |  |  | |
| Exclude patients with serum potassium values outside below 5 mEq/L or above 8 mEq/L | 18,248 | 1,810 | 18,248 | |
| **Step 10** |  |  |  | |
| **Patiromer Only** | **6,480** |  | **6,480** | |
| **SZC Only** | **11,768** |  | **11,768** | |

**Table S2. Charlson-Deyo comorbidities and related ICD-10-CM diagnosis and procedure codes**

| **Comorbidity** | **Type** | **Deyo’s Algorithm with ICD-10-CM codes** |
| --- | --- | --- |
| Myocardial infarction | Dx | I21.x, I22.x, I23.x, I25.2 |
| Congestive heart failure | Dx | I50.x |
| Peripheral vascular disease | Dx | I73.9, I71.00, I71.01, I71.02, I71.03, I71.1, I71.2, I71.3, I71.4, I71.5, I71.6, I71.8, I71.9, I96, Z95.828 |
|  | Proc | 04RK07Z, 04RK0JZ, 04RK0KZ, 04RK47Z, 04RK4JZ, 04RK4KZ, 04RL07Z, 04RL0JZ, 04RL0KZ, 04RL47Z, 04RL4JZ, 04RL4KZ, 04RM07Z, 04RM0JZ, 04RM0KZ, 04RM47Z, 04RM4JZ, 04RM4KZ, 04RN07Z, 04RN0JZ, 04RN0KZ, 04RN47Z, 04RN4JZ, 04RN4KZ, 04RP07Z, 04RP0JZ, 04RP0KZ, 04RP47Z, 04RP4JZ, 04RP4KZ, 04RQ07Z, 04RQ0JZ, 04RQ0KZ, 04RQ47Z, 04RQ4JZ, 04RQ4KZ, 04RR07Z, 04RR0JZ, 04RR0KZ, 04RR47Z, 04RR4JZ, 04RR4KZ, 04RS07Z, 04RS0JZ, 04RS0KZ, 04RS47Z, 04RS4JZ, 04RS4KZ, 04RT07Z, 04RT0JZ, 04RT0KZ, 04RT47Z, 04RT4JZ, 04RT4KZ, 04RU07Z, 04RU0JZ, 04RU0KZ, 04RU47Z, 04RU4JZ, 04RU4KZ, 04RV07Z, 04RV0JZ, 04RV0KZ, 04RV47Z, 04RV4JZ, 04RV4KZ, 04RW07Z, 04RW0JZ, 04RW0KZ, 04RW47Z, 04RW4JZ, 04RW4KZ, 04RY07Z, 04RY0JZ, 04RY0KZ, 04RY47Z, 04RY4JZ, 04RY4KZ |
| Cerebrovascular disease | Dx | I60.x, I61.x, I62.x, I63.x, I65.x, I66.x, I67.x, I68.x, I69.x, G45.x |
| Dementia | Dx | F03.90, F01.50, F01.51, F03.91, F02.80, F02.81 |
| Chronic pulmonary disease | Dx | J40, J41.0, J41.1, J44.9, J44.0, J41.8, J42, J43.9, J45.20, J45.21, J45.22, J44.1, J45.990, J45.991, J45.909, J45.998, J45.902, J45.901, J47.9, J47.1, J67.0, J67.1, J67.2, J67.3, J67.4, J67.5, J67.6, J67.7, J67.8, J67.9, J60, J61, J62.8, J63.0, J63.1, J63.2, J63.3, J63.4, J63.5, J63.6, J66.0, J66.1, J66.2, J66.8, J64, J68.4 |
| Rheumatic disease | Dx | M32.10, M34.0, M34.1, M34.9, M33.20, M06.9, M05.00, M05.30, M05.60, M06.1, M05.10, M35.3 |
| Peptic ulcer disease | Dx | K25.0, K25.1, K25.2, K25.3, K25.4, K25.5, K25.6, K25.7, K25.9, K26.0, K26.1, K26.2, K26.3, K26.4, K26.5, K26.6, K26.7, K26.9, K27.0, K27.1, K27.2, K27.3, K27.4, K27.5, K27.6, K27.7, K27.9, K28.0, K28.1, K28.2, K28.3, K28.4, K28.5, K28.6, K28.7, K28.9 |
| Mild liver disease | Dx | K70.30, K73.9, K73.0, K75.4, K73.2, K73.8, K74.0, K74.60, K74.69, K74.3, K74.4, K74.5 |
| Diabetes without chronic complication | Dx | E11.9, E10.9, E13.9, E11.65, E10.65, E13.65, E10.1x, E11.1x, E13.1x, E11.0x, E13.0x, E11.64x, E10.64x, E13.64x |
| Diabetes with chronic complication | Dx | E10.2x, E10.3x, E10.4x, E10.5x, E10.61x, E10.62x, E10.63x, E10.69, E10.8, E11.2x, E11.3x, E11.4x, E11.5x, E11.61x, E11.62x, E11.63x, E11.69, E11.8, E13.2x, E13.3x, E13.4x, E13.5x, E13.61x, E13.62x, E13.63x, E13.69, E13.8 |
| Hemiplegia or paraplegia | Dx | G04.1, G11.4, G80.1, G80.2, G81.x,  G82.x, G83.0, G83.1x, G83.2x, G83.3x, G83.4, G83.9 |
| Moderate or severe renal disease | Dx | I12.0, I13.11, I13.2, N03.2, N03.3, N03.5, N03.8, N03.9, N05.2 N05.5, N05.9, N08, N18.x, N19.x, N25.x, Z49.0x, Z49.3x, Z94.0, Z99.2 |
| Any malignancy, including lymphoma and leukemia, except malignant neoplasm of skin | Dx | C00.x-C75.x (except C43.x and C44.x), C81.x-C85.x, C88.x, C90.x, C91.x-C95.x, C96.x, C7A.xxx, C7B.xxx, D00.xx, D01.xx, D02.x, D03.xx, D05.xx, D06.x, D07.xx, D09.xx, D47.9 |
| Moderate or severe liver disease | Dx | I85.00, I85.01, I85.10, I85.11, I86.4, K70.4x, K71.1x, K71.7, K72.xx, K76.6, K76.7 |
| Metastatic solid tumor | Dx | C77.x, C78.x, C79.x, C80.0 |
| HIV disease | Dx | B20 |

| **Table S3. Definition of other comorbidities and kidney-related outcomes** | | | |
| --- | --- | --- | --- |
|  | | | |
| **Condition** | **Type** | **ICD-10-CM Codes** | **CPT-4 Codes** |
| Hyperkalemia | Dx | E87.5 |  |
| Acute Kidney Injury (AKI) | Dx | N17.% |  |
| Cardiac arrhythmia | Dx | I48%, I49% |  |
| Hemodialysis and  Peritoneal dialysis | Dx | R88.0, T81.502x, T81.512x, T81.522x, T81.532x, T81.592x, T82.41x, T82.42x, T82.43x, T82.49x, T85.611x, T85.621x, T85.631x, T85.691x, T85.71x, Y62.2, Y84.1, Z49.01, Z49.02, Z49.31, Z49.32, Z99.2 | 90935, 90937, 90939-90945, 90947, 90976-90985, 90988-90998 |
|  | Proc | B50W%, B5W%, 5A1D%, 3E1M39Z |  |
| **Chronic kidney disease** | Dx |  |  |
| *CKD Stage 1* | Dx | N18.1%, |  |
| *CKD Stage 2* |  | N18.2% |  |
| *CKD Stage 3* |  | N18.3% |  |
| *CKD Stage 4* |  | N18.4% |  |
| *CKD Unspecified* |  | N18.9% |  |
| **End-stage renal disease** |  |  |  |
| *CKD Stage 5* | Dx | N18.5%, I12.0,I13.2 |  |
| End stage renal disease | Dx | N18.6% |  |
| Heart failure | Dx | I50.%, I11.0, I13.0, I13.2 |  |
| COVID-19 | Dx | U07.1 |  |
| Hypertension |  | I10.%, I11.%, I12.%, I13.%, I15.%, I16.% |  |
| Ischemic heart disease | Dx | I20.x, I21.x, I22.x, I23.x, I24.x, I25.x, Z95.1, Z98.61 |  |
| Morbid obesity | Dx | E66.01, E66.2, Z68.4% |  |
| Trauma |  | S06.5X9A, 506.6X9A, S06.5X0A,S06.6X0A, S27.2XXA,S06.5X7A, S27.0XXA, S06.6X1A, S06.2X9A, S06.5X1A, S06.6X7A, S06.6X6A, S06.369A, S06.5X6A,S06.5X2A,S06.309A, S06.5X5A, S06.349A, S27.1XXA, S06.2X6A, S06.6X2A, S06.1X9A, S06.350A, S06.360A, T79.4XXA, S06.2X0A, S06.340A, S06.359A, S06.2X7A, S06.300A, S06.5X3A, S06.6X4A, S06.1X7A, S06.351A, 538.1XXA, R56.1, S06.2X3A, S06.316A, S06.341A, S06.356A, S06.5X4A, S06.6X5A, S06.6X8A, S06.6X9D, S88.021A, S88.122A, S06.1X1A, S06.1X6A, S06.2X4A, S06.346A, S06.355A, S06.360S, S06.361A, S06.366A, S06.367A, S06.386A, S06.5X9D, S06.5X9S, S06.6X1D, S06.6X3A, S12.330A, S12.530A, S12.550A,S28.0XXA, S33.4XXA, S58.121A,S78.111A, S88.111A, S88.112A, S98.022A,T79.6XXA,T79.7XXA, T79.8XXA, T79.A12A,T79.A22A |  |
| Burn |  | T20%, T21%, T22%, T23%, T24%, T25%, T26%, T27%, T28%, T29%, T30%, T31%, T32% |  |
| Crash |  | V00%-V99% |  |
| COVID-19 |  | U07.1 |  |
